# Reduction in adipose tissue volume using a new high-power radiofrequency technology combined with infrared light and mechanical manipulation for body contouring

**DOI:** 10.1007/s10103-014-1564-x

**Published:** 2014-04-01

**Authors:** Maurice A. Adatto, Robyn M. Adatto-Neilson, Grietje Morren

**Affiliations:** 1 Skinpulse Dermatology, Laser & Beauty Centers, Geneva, Switzerland; 2 Private Practice, Leuven, Belgium

**Keywords:** VelaShape II, Circumferential reduction, Body contouring, Skin tightening

## Abstract

A growing patient demand for a youthful skin appearance with a favorable body shape has led to the recent development of new noninvasive body contouring techniques. We have previously demonstrated that the combination of bipolar radiofrequency (RF) and optical energies with tissue manipulation is an efficient reshaping modality. Here, we investigated the efficacy and safety of a new high-power version of this combined technology, in terms of adipose tissue reduction and skin tightening. Thirty-five patients received one treatment per week over 6 weeks to their abdomen/flank, buttock, or thigh areas and were followed up to 3 months post completion of the treatment protocol. This new device has an increased power in the bipolar RF, as this parameter appears to be the most important energy modality for volume reduction. Patient circumferences were measured and comparisons of baseline and post treatment outcomes were made. Diagnostic ultrasound (US) measurements were performed in 12 patients to evaluate the reduction in adipose tissue volume, and a cutometer device was used to assess improvements in skin tightening. We observed a gradual decline in patient circumferences from baseline to post six treatments. The overall body shaping effect was accompanied with improvement in skin tightening and was clearly noticeable in the comparison of the before and after treatment clinical photographs. These findings correlated with measurements of adipose tissue volume and skin firmness/elasticity using diagnostic US and cutometer, respectively. The thickness of the fat layer showed on average a 29 % reduction between baseline and the 1-month follow up. The average reduction in the circumference of the abdomen/flanks, buttocks, and thighs from baseline to the 3-month follow-up was 1.4, 0.5, and 1.2 cm, respectively, and 93 % of study participants demonstrated a 1–60 % change in fat layer thickness. Patients subjectively described comfort and satisfaction from treatment, and 97 % of them were satisfied with the results at the follow-up visit. The application of high-power RF energy combined with infrared (IR), mechanical massage, and vacuum appears to be an effective modality for the reduction in circumferences of the abdomen/flank, buttock and thigh regions, and the improvement of skin appearance. The present study performed with a new device suggests that the underlying mechanism of action is reduction in the subcutaneous adipose tissue volume and intensification of dermal matrix density.

## Introduction

Localized subcutaneous fat deposits and tissue laxity are of growing concern among cosmetic patients, the contributing factors of which include chronological aging, photoaging, as well as changes in body dimensions due to pregnancy and significant weight loss. The most popular body contouring approaches used to improve the cosmesis of subcutaneous fat deposits and skin laxity are surgical and include liposuction, abdominoplasty, and thigh lifts, among other procedures. However, in tandem with cosmetic patients’ desire for a favorable body shape is their increasing demand for noninvasive treatment approaches that are painless, safe, and require little to no downtime. This increasing demand has led to the rapid growth and development of noninvasive, nonsurgical treatment techniques. Although surgical techniques can result in the most pronounced outcomes in respect to improved body contouring results, they are also associated with inherent risks as well as prolonged recovery times. These factors, in combination with today’s cosmetic patients’ active lifestyles and coupled with their desire for noninvasive treatment options have further popularized noninvasive, nonsurgical treatment approaches.

The first most common and available noninvasive treatments for body contouring were based on nonthermal mechanical rollers and suction systems that were thought to cause vasodilatory effects, which enhance lymphatic drainage in fat deposits and improve microcirculation. Over the past couple of years, however, the technology has moved towards the use of thermal-based suction devices which combine radiofrequency (RF) with or without infrared energies and mechanical massage. The application of energy to the skin’s surface produces heat in the dermis and subcutaneous tissues with subsequent induction of collagen denaturation and neocollagenesis, resulting in tissue tightening [1–4]. The RF technology delivers a thermal stimulus to the skin and superficial adipose tissue causing a thickening of the dermis and enhancement of fat cell metabolism, resulting in a reduction in skin laxity and adipocyte volume [1–5].

The VelaShape device (Syneron Medical Ltd. Yokneam, Israel) incorporates four treatment modalities including pulsed vacuum and mechanical massage, bipolar RF energy, and IR light, the latter of which preheats the targeted tissue, mitigating impedance, and thereby allowing greater attraction of the RF current and deeper penetration of RF energy into the targeted tissues [1–6]. This study evaluated the efficacy and safety of the VelaShape II device (Syneron Medical Ltd., Yokneam, Israel), a new high-power version of its predecessor the VelaShape, in terms of adipose tissue reduction and skin tightening.

## Materials and methods

In this prospective, two-center clinical trial conducted at Skinpulse Dermatology and Laser Centre, Geneva, Switzerland, and Dr. Morren’s private practice, Leuven, Belgium, treatment was performed using the VelaShape II system, a device that combines four different technologies including broadband IR (infrared), bipolar RF-pulsed vacuum and massage rollers. The broadband IR light spectrum is 700–2,000 nm with a high pass filter, at up to 35 W. The RF frequency is 1 MHz and up to 60 W. Pulsed vacuum was set up at 200 mbar of negative pressure.

The combination of the IR and pulsed vacuum coupled RF technologies causes a deep heating of the connective tissue including the fibrous septae. This in turn promotes an increase in collagen deposition and cellular metabolism resulting in a localized reduction in skin laxity and volume [1–4]. The additional mechanical tissue manipulation by the vacuum and massage rollers, causes an immediate increase in the local circulation and enhances lymphatic drainage, both effects of which are considered to be essential components for healthy skin structure. The VelaShape II system has two applicators, namely the Vsmooth with a 40 mm × 40 mm spot size and the Vcontour with a 30 mm × 30 mm spot size. The applicators are fitted with a replaceable cap that has a treatment chamber, into which the targeted skin is repeatedly drawn during treatment via mechanical manipulation and is exposed to IR and RF energies. The user can individualize treatment by adjusting the energies and vacuum levels according to the patient and anatomical site treated.

The study included 35 healthy adult female patients who were between 21 and 58 years of age (mean age 43) with clinically appreciable skin laxity and localized subcutaneous fat deposits on the abdomen/flanks, buttocks or thigh regions, and Fitzpatrick skin types I to III. Patient inclusion criteria were the presence of at least 20 mm of subcutaneous fat (assessed by ultrasound) and the presence of lax skin and cellulite. Study exclusion criteria were mainly pregnancy, lactation, and any kind of previous cosmetic treatment in these areas for the last 12 months. Every patient signed the informed consent prior to the study. Study participants were treated for circumferential reduction on the abdomen/flanks (*n* = 32), buttocks (*n* = 14), and thighs (*n* = 16) and received a total of six treatments performed once or twice a week. All of the treatments in this study were performed with the Vsmooth large spot applicator using RF energy of 60 W and IR energy of up to 35 W. Each procedure was performed using the established and standardized treatment protocol of the VelaShape II system, which at the time of this study was a new device with increased power. The pulsed vacuum was typically set at level 2 (200 mbar of negative pressure). Treatment sessions typically lasted from 35 to 45 min, in which the goal was to treat the area until the target tissue temperature of between 39–41 °C has been reached and maintain it for at least 5 min per 10 × 10 cm^2^ zone (per the VelaShape II user manual). The temperature maintained was measured at skin level using an external thermometer. It has been shown that in the temperature range 37–44 C, skin blood perfusion increases 10 times, muscle about nine times, and fat only two times [13]. The skin cools much faster than the fat because of the increased blood flow, thus the temperature is maintained much longer in the fat layer than what we measure on the skin.

Patients did not gain or lose weight significantly during the course of the study, as they were weighed at each visit. Various clinical evaluations were performed by an independent observer at baseline, after the fourth treatment, immediately after the last treatment, and 1 and 3 months after the last treatment. Objective clinical assessments included changes in the fat layer thickness and skin firmness/elasticity, which were performed using an external ultrasound probe (Echoblaster 128, Telemed Ltd., Vilnius, Lithuania) and a cutometer, respectively. Both the ultrasound and cutometer technologies used to ascertain the objective clinical changes achieved in this study are FDA-approved modalities. Measurements were taken in three consecutive repetitions, and the average score (in mm) was recorded. Improvement evaluation was performed by the physician using the following percentile categories: 0, 1–24, 25–49, 50–74, and 75–100 %. All clinical photographs were taken with a medical standardized system (Profect Full Body System, Profect Medical Technologies LLC, Pound Ridge, NY, USA). Patients evaluated treatment outcome satisfaction based on the following satisfaction scale: not satisfied, slightly satisfied, satisfied, very satisfied, and extremely satisfied. Safety and patients’ report of treatment-associated sensation was monitored throughout the study.

## Results

All of the patients met the inclusion/exclusion criteria of the study and signed an informed consent for US and cutometer measurements prior to its initiation. Results showed that all of the 35 healthy adult women (aged 21 to 58 years, mean 43 years) completed the clinical trial. The Fitzpatrick skin type allocation was 9 % type I, 85 % type II, and 6 % type III. The 35 patients included in the study received VelaShape II treatment to one or more anatomical sites including the abdomen/flanks (*n* = 32), buttocks (*n* = 14), and thighs (*n* = 16). The average reduction in the circumference of the abdomen/flanks, buttocks, and thighs from baseline to the 3-month follow-up was 1.4, 0.5, and 1.2 cm, respectively, while the SD was 2, 0.7, and 3.3 cm, respectively. *P* value (Wilcoxon signed rank test for single group median) was 0.0004, 0.0446, and 0.1503, respectively (Table 1).

**Table 1:** Circumference 3 months after end of treatments

	Delta circ. thigh	Delta circ. abdomen	Delta circ. buttock
Mean	1.209375	1.428125	0.475468
S.E.M.	0.8339692	0.362374	0.195029
SD	3.3358766	2.049899	0.729733
Variance	11.128073	4.202087	0.532511
Coef. var.	2.7583476	1.435378	1.534768
Minimum	−1.5	−3.5	−0.5
Maximum	13	6	2
Sum	19.35	45.7	6.656557
*N*	16	32	14
*P* value (Wilcoxon signed rank test for single group median)	0.1504513	0.000365	0.044603
*P* value (Kruskal-Wallis test)	0.1267881158		

The thickness of the fat layer as per the comparative ultrasound measurements taken showed on average a 29 % reduction between baseline and the 1-month follow-up, and 93 % of study participants demonstrated a 1–60 % change in fat layer thickness (Fig. 3).

**Fig. 1:**
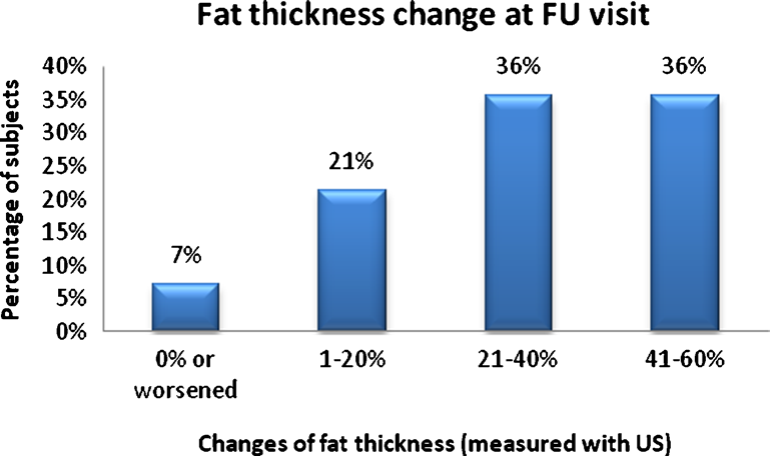
Fat thickness change at FU visit

The physician’s evaluation measurements revealed that almost all patients had some level of overall improvement in adipose tissue reduction and skin tightening in the treated areas, with 60 % of patients showing a 1–24 % improvement, 27 % showing a 25–49 % improvement, and 5 % showing a 50–74 % improvement, while only 8 % of patients showed no improvement in the treated anatomical sites (Fig. 1). At the final follow-up visit, 97 % of patients expressed an overall satisfaction with the treatment outcomes, including 3 % being extremely satisfied, 32 % very satisfied, 52 % satisfied, and 10 % slightly satisfied. The overwhelming majority of patients tolerated the treatments well, with 69 % of patients subjectively rating the treatment as very comfortable, 21 % comfortable, 6 % somewhat comfortable, and 3 % somewhat uncomfortable, while only 3 % perceived the treatment as very uncomfortable. Side effects of treatment included transient erythema and edema in all patients and two patients with bruising at the treated site. Adverse events included a burn to the abdomen in one patient, which could be treated with remedial therapy.

## Discussion

**Fig. 2:**
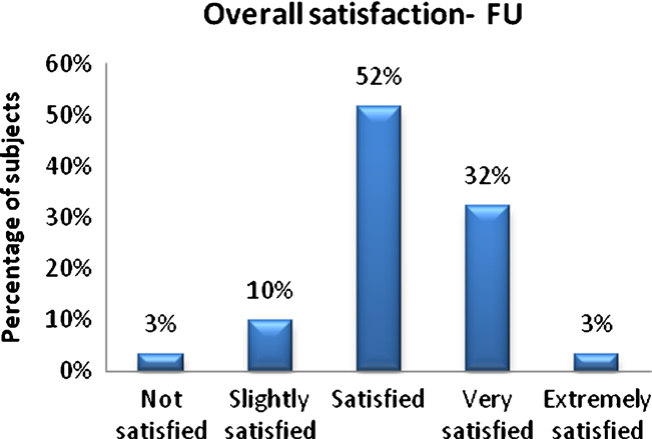
Overall satisfaction—FU

This study supports the safety and efficacy of the VelaShape II system for the improvement in circumferential reduction and skin laxity in the abdomen/flanks, buttocks, and thigh regions (Fig. 2). The safety and efficacy of an older version of the device (VelaSmooth, Syneron Medical Ltd., Yokneam, Israel) has already been demonstrated in previous clinical trials in terms of circumference reduction and cellulite improvement, mainly in thighs and buttocks [6–11]. The VelaShape (immediate predecessor to the VelaShape II) was the first device to achieve an FDA indication for circumferential reduction in 2007, following clinical trials performed on thighs [1, 6, 9–14].

**Fig. 3:**
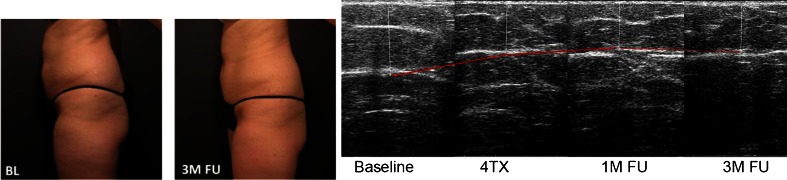
Four treatment results, reduction of fat thickness is visible and stable at 1 and 3 months FU

The clinical effect seen with VelaShape treatment is thought to occur as follows: the pulsed vacuum combined with the action of massage rollers causes a vasodilation as well as an increased local circulation and lymphatic drainage in the treated area. The subsequent increase in available oxygen can facilitate an increase in the localized fat metabolism. In addition, the heat generated from the application of IR and RF energies theoretically increases the available oxygen, further enhancing fat metabolism and causes the adipocytes to shrink as they break down the fat. The mechanical massage also enhances the flow of these breakdown products to the lymphatic system and stretches the fibrous septae. The collagen fibers in the dermis and fibrous septae shrink, resulting in smaller fat chambers and skin tightening. The heat energy also causes collagen shrinkage and stimulates the fibroblasts to produce new collagen fibers. Taken together, these combined actions result in circumferential reduction and improvements in skin laxity and the appearance of cellulite (Fig. 3).

Among other changes, one of the pivotal upgrades made to the VelaShape II device from its immediate predecessor is an increase in power in the bipolar RF energy by 20 % to 60 W. The increased power in the bipolar RF energy appears to be the most important energy modality that can impact and enhance volume reduction. The 40 mm × 40 mm Vsmooth large spot applicator used in this study can emit RF energy of 60 W and IR of up to 35 W. The simultaneous application of IR and RF heat energies is combined with mechanical tissue manipulation achieved with massage rollers and pulsed, the latter of which allows for a higher penetration depth for higher peak heating of the subcutaneous tissue.

The peak temperature reached in the tissues and the time over which that temperature is maintained are two important factors that impact tissue tightening. Achieving and maintaining a peak temperature over a period of time result in a progressive skin tightening and subsequent measureable reductions in circumference [15]. It is widely known that clinical results are dependent on the maintenance of targeted tissue temperature over 40 °C [2, 14]. Therefore, one of the goals during treatment with the device in this study was to reach and maintain a target tissue temperature between 40–41 °C for at least 5 min per 10 × 10 cm^2^ zone. Data has shown that at 10- and 15-mm tissue depths, the temperature elevates after VelaShape treatment and is maintained over 40 °C, long after completion of treatment enabling an enhanced thermal effect for a more effective clinical outcome [14]. It is thought that the synergy of the combined technologies of IR and RF energies (heating the tissue up to 3 and 15 mm, respectively), as well as vacuum and massage rollers can result in improved results that may last longer compared to previous versions of the device. The 20 % higher bipolar RF energy of the VelaShape II device up to 60 W enables a deeper and more intense heating of the dermis and subcutaneous tissues, allowing for clinicians to reach the target temperature quicker, resulting in a reduction of treatment times, particularly when compared to earlier versions of the device.

Limitations of this study include a relatively small patient cohort as well as the lack of extended follow-up evaluations and untreated control subjects. In addition, each abdomen measurement could ideally be taken at the same point in the respiratory cycle in order to better address measurement bias. Moreover, the timing of measurements in respect to the patients’ menses (i.e., bloating) could potentially alter circumference measurements. One can also assume that large meals or volumes of fluid taken directly prior to circumference measurement can affect the measurement result [16]. Future clinical trials that can address these points could be part of the focus of future clinical trials, which may further support the clinical outcomes achieved here.

## Conclusion

In conclusion, the combination of bipolar RF, IR light, and mechanical tissue manipulation with pulsed vacuum and massage rollers appears to be a safe and effective therapeutic modality for the reduction of adipose tissue volume and skin tightening. It is suggested that the 20 % higher bipolar RF energy available in the VelaShape II device may also result in a more intense heating of the targeted tissues, resulting in both faster treatment times as well as improved clinical outcomes.

## References

[1] Sadick NS (2008). Tissue tightening technologies: fact or fiction. Aesthet Surg J.

[2] Zelickson B, Kist D, Berstein E, Brown D, Ksenzenko S, Burns J, Kilmer S, Mehregan D, Pope K (2004). Histological and ultrastructural evaluation of the effects of a radiofrequency-based nonablative dermal remodeling device. A pilot study. Arch Derm.

[3] Sadick NS (2005). Combination radiofrequency and light energies: electro-optical synergy technology in esthetic medicine. Derm Surg.

[4] Elsaie ML (2009). Cutaneous remodeling and photorejuvenation using radiofrequency devices. Indian J Dermatol.

[5] Alster TS, Lupton JR (2007). Nonablative cutaneous remodeling using radiofrequency devices. Clin Dermatol.

[6] Alster TS, Tanzi EL (2005). Cellulite treatment using a novel combination radiofrequency, infrared and mechanical tissue manipulation device. J Cosmet Laser Ther.

[7] Alster TS, Tehrani M (2006). Cellulite treatment using a novel combination radiofrequency, infrared light, and mechanical tissue manipulation device. Lasers Surg Med.

[8] Sadick NS, Magro C (2007). A study evaluating the safety and efficacy of the VelaSmooth system in the treatment of cellulite. J Cosmet Laser Ther.

[9] Kulick M (2006). Evaluation of the combination of radio frequency, infrared energy and mechanical rollers with suction to improve skin surface irregularities (cellulite) in a limited treatment area. J Cosmet Laser Ther.

[10] Sadick NS, Mulholland RS (2004). A prospective clinical study to evaluate the efficacy and safety of cellulite treatment using the combination of optical and RF energies for subcutaneous tissue heating. J Cosmet Laser Ther.

[11] Wanitphakdeedecha R, Manuskiatti W (2006). Treatment of cellulite with a bipolar radiofrequency, infrared heat, and pulsatile suction device: a pilot study. J Cosmet Dermatol.

[12] Nootheti PK, Magpantay A, Yosowitz G, Calderon S, Goldman M (2006). A single center, randomized, comparative prospective clinical study to determine the efficacy of the Velasmooth System versus the Triactive System for the treatment of cellulite. Lasers Surg Med.

[13] Drizdal T, Togni P, Viseki L, Vrba J (2010). Comparison of constant and temperature dependent blood perfusion in temperature prediction for superficial hyperthermia. Radioengineering.

[14] Mulholland RS (2004). Bipolar radiofrequency, infrared heat and pulsatile suction in the non-surgical treatment of focal lipodystrophy and cellulite. Aust Cosmet Surg.

[15] Brightman L, Weiss E, Chapas AM, Karen J, Hale E, Bernstein L, Geronemus RG (2009). Improvement in arm and post-partum abdominal and flank subcutaneous fat deposits and skin laxity using a bipolar radiofrequency, infrared, vacuum and mechanical massage device. Lasers Surg Med.

[16] Agarwal SK, Misra A, Aggarwal P, Bardia A, Goel R, Vikram AK, Wasir JS, Hussain N, Ramachandran K, Pandey RM (2009). Waist circumference measurement by site, posture, respiratory phase, and meal time: implications for methodology. Obes J.

